# Clinical experience of primary subcutaneous mycoses in Shanghai: a retrospective analysis

**DOI:** 10.3389/fcimb.2025.1520608

**Published:** 2025-03-18

**Authors:** Qian Yu, Lulu Li, Yuanyuan Wang, Zhiqin Gao, Siyu Liu, Jingwen Tan, Xiaoping Liu, Wei Li, Lianjuan Yang

**Affiliations:** ^1^ Department of Medical Mycology, Shanghai Skin Disease Hospital, Tongji University School of Medicine, Shanghai, China; ^2^ Department of Medical Cosmetology, Shanghai Skin Disease Hospital, Tongji University School of Medicine, Shanghai, China

**Keywords:** primary subcutaneous mycoses, clinical experience, diverse mycological species, therapeutic schedule, long-term follow-up

## Abstract

**Introduction:**

Primary subcutaneous mycoses are a heterogeneous group of fungal infections caused by pathogenic or opportunistic organisms. In recent years, cases have steadily increased in Shanghai, an area where the disease was previously uncommon. This study aimed to summarize clinical experiences with primary subcutaneous mycoses in Shanghai, to optimize their management.

**Method:**

A retrospective analysis was conducted at Shanghai Dermatology Hospital from January 2018 to March 2023 and enrolled 33 patients with confirmed primary subcutaneous mycoses. Their medical histories, clinical features, histopathological findings, etiological characteristics, drug sensitivity tests, therapeutic regimens, and follow-up data were recorded.

**Results:**

Identification of pathogens from skin tissue cultures revealed distinct colonial morphologies across diverse mycological species. The isolates included yeast (45.5%), mold (30.3%), and dimorphic fungi (24.2%). The most common species were *C. parapsilosis* (n = 8, 24.2%), *T. rubrum* (n = 5, 15.2%), and *S. schenckii* (n = 8, 24.2%). Thirty-two patients received systemic antifungal treatment based on the results of the drug sensitivity test, whereas one patient was treated with complete surgical resection, owing to a single plaque. Post-treatment surveillance was important for the effective management of the condition.

**Conclusion:**

This study highlights the considerable diversity among fungal species implicated in primary cutaneous mycoses and underscores the complexities involved in their accurate diagnosis and management. Correcting unhealthy lifestyles, boosting immunity, and completely removing and avoiding re-exposure to the pathogenic fungi can effectively reduce the risk of relapse in primary subcutaneous mycoses. Our findings provide valuable insights into primary subcutaneous mycoses and may contribute to improved patient prognoses.

## 1 Introduction

Primary subcutaneous mycoses comprise a heterogeneous group of fungal infections caused by pathogenic or opportunistic organisms, ranging from yeasts and molds to dimorphic fungi (Arenas et al., 2012). These infections primarily affect cutaneous and subcutaneous tissues but can occasionally spread to other sites, leading to disseminated or systemic infections (La Hoz and Baddley, 2012; Elgart, 2014; Carrasco-Zuber et al., 2016). Their clinical course may be chronic, treatment-resistant, or even life-threatening and is often associated with high morbidity and mortality (Pappas et al., 2010; Brown et al., 2012). However, estimates of the global prevalence of primary subcutaneous mycoses have rarely been reported. This is partly due to their rarity and diagnostic challenges (Nishikawa, 2015).

Primary subcutaneous mycoses are more prevalent in certain regions and among specific types of employment (Bonifaz et al., 2010). For example, primary subcutaneous mycoses, including sporotrichosis, chromoblastomycosis, and phaeohyphomycosis, are prevalent in subtropical and tropical regions (Barros et al., 2011; Queiroz-Telles et al., 2009). With respect to occupational exposure risk groups, these mycoses are mainly concentrated among farmers, gardeners, miners, and pottery workers. The affected population is mostly adults aged 20–60 years (Barros et al., 2011; Guevara et al., 2021). They are relatively less common in Shanghai, China. However, in recent years, reports of these cases have steadily increased in Shanghai, likely because of the increasing number of patients with immune deficiencies or those receiving immunosuppressive therapy. Clinical manifestations vary, including papules, plaques, nodules, and ulcers, which may present as single or multiple skin lesions (La Hoz and Baddley, 2012; Elgart, 2014; Carrasco-Zuber et al., 2016). The skin lesion presentation patterns depend on host-related factors, the type of fungal organism, and the mode of transmission (Queiroz-Telles et al., 2003). The nonspecific clinical symptoms of primary subcutaneous mycoses pose significant challenges for clinicians. Increased awareness of these infections can help prevent adverse outcomes associated with delayed diagnosis and treatment. Therefore, a study that focuses on primary subcutaneous mycoses in Shanghai and summarizes the clinical experience is warranted.

In this study, we retrospectively analyzed data from patients with primary subcutaneous mycoses who were diagnosed and treated at Shanghai Dermatology Hospital from January 2018 to March 2023. We summarized the clinical features, histopathological findings, etiological characteristics, drug sensitivity test results, therapeutic regimens, and follow-up data to optimize the management of subcutaneous mycoses in Shanghai.

## 2 Materials and methods

### 2.1 Clinical sample collection

This retrospective study was conducted at Shanghai Dermatology Hospital, China. We enrolled patients who were admitted to the hospital between January 2018 and March 2023 and were suspected of having deep dermatophytosis. The study was reviewed and approved by the Ethics Committee of Shanghai Dermatology Hospital. Written informed consent was obtained from all participants for inclusion in the study and the publication of any potentially identifiable images or data. Patient details, including age, sex, signs and symptoms of the disease, medical history, contributing factors, therapeutic schedule, and prognosis, were documented. Clinical samples were collected from patients and underwent hematological and blood chemistry evaluations, direct microscopic examination for the presence of fungi and bacteria, skin biopsy with special staining, skin tissue culture, and molecular identification of microbial isolates. Skin tissue specimens (6×6×6 mm^3^) were divided into two sections for histopathological investigation and tissue culture (Brodell, 2021). Positive tissue culture colonies were further identified through morphological and molecular analyses (Quindós et al., 2012). Contaminated or extremely small skin tissue specimens were excluded from the study.

**Figure 1:**
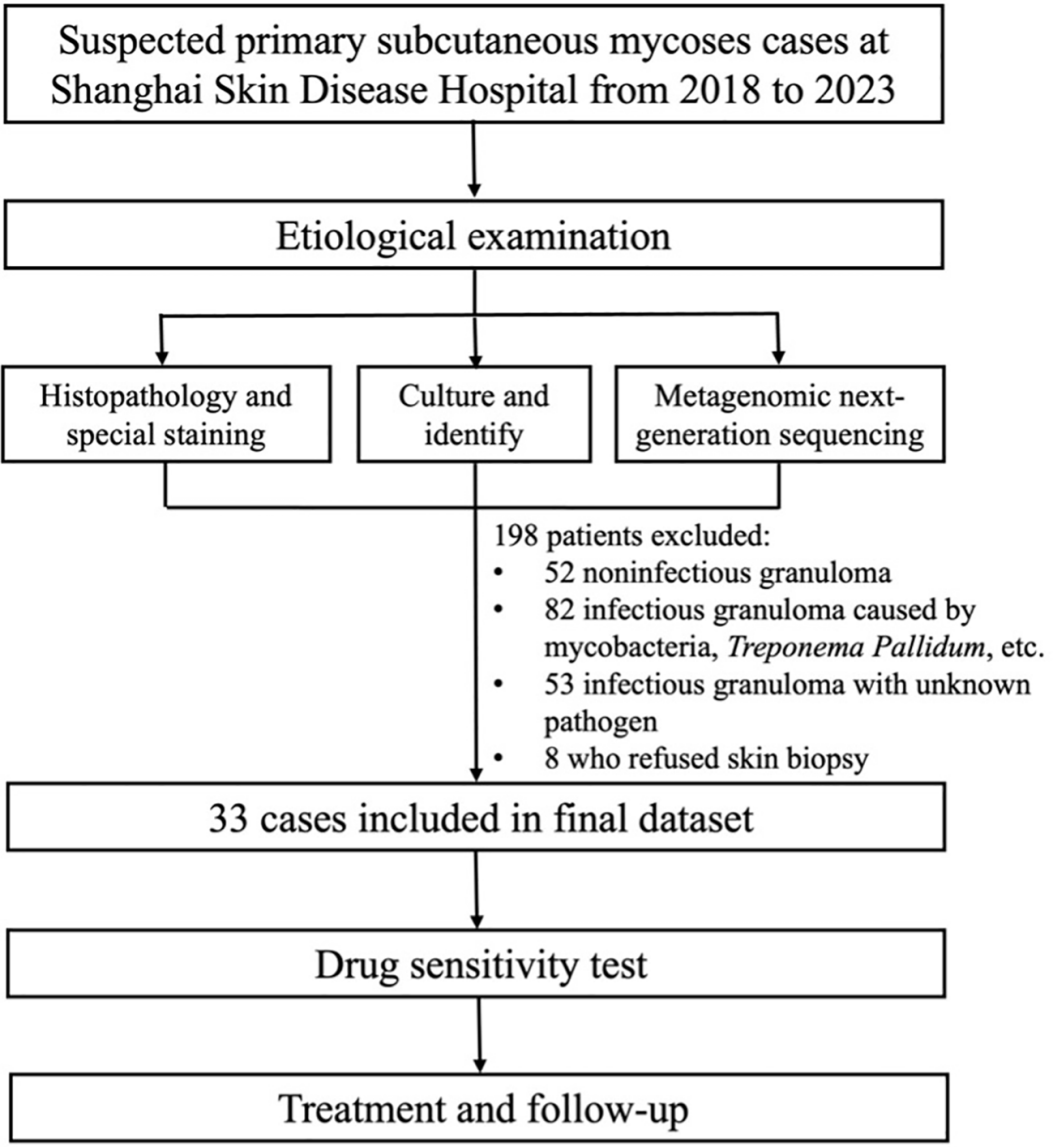
Flow diagram of the study.

A total of 231 patients suspected of having primary subcutaneous mycoses were recruited. Among them, 52 patients were diagnosed with noninfectious granulomas, 82 were diagnosed with infectious granulomas caused by non-fungal pathogens such as *Mycobacterium* and *Treponema pallidum*, and 53 had infectious granulomas of unknown origin. Additionally, eight patients refused skin biopsy. Finally, 33 patients were included in the study. We conducted drug susceptibility tests on the pathogenic fungi and subsequently provided corresponding treatment recommendations, followed by patient follow-up (**Figure 1**).

### 2.2 Culture and identification

Skin tissues were excised, ground, and decontaminated using 2% *N*-acetyl-L-cysteine sodium hydroxide (pH 6.8) following a standard protocol (Gonzalez Santiago et al., 2014). The skin tissue homogenates were prepared for standby application by centrifuging the pathogen suspended in phosphate-buffered saline at 3,000×g for 15 min. The concentrated sediment was then resuspended in 0.5 mL of sterile water. The homogenates were cultured on Sabouraud dextrose agar (SDA) at 26°C and 35°C and in Lowenstein-Jensen medium at 30°C and 37°C in 5% CO_2_. After the initial genus classification, the fungus was subcultured in a different medium. Following the adequate growth of the fungal isolates, the pathogenic species were identified based on macroscopic and microscopic characteristics.

Subsequently, fungus genomic DNA was extracted from the colonies and amplified using polymerase chain reaction (PCR) using the fungal primers, internal transcribed spacer ITS1 and ITS4. (ITS1: 5′-TCCGTAGGTGAACCTGCGG-3′. ITS4: 5′-TCCTCCGCTTATTGATATGC-3′). PCR products showing electrophoretic bands were sequenced, and the species was identified using BLAST analysis (http://www.ncbi.nlm.nih.gov/BLAST/). Based on sequencing data and morphological characteristics, these fungi were categorized into three distinct groups: yeast and yeast-like fungi, mold fungi, and dimorphic fungi.

### 2.3 Diagnostic criteria

In this study, we defined primary subcutaneous mycoses as fungal infections localized to the skin and subcutaneous tissue, without any evidence of systemic infections (Carrasco-Zuber et al., 2016). A patient was diagnosed with primary subcutaneous mycoses if they met all the following diagnostic criteria: clinical suspicion of the infection; skin biopsy showing infectious granuloma, irrespective of periodic acid-Schiff (PAS) and Grocott methenamine silver (GMS) staining; and a positive skin tissue culture confirming the diagnosis. Exclusion criteria included superficial fungal infections confined to the stratum corneum, hair, or nails; evidence of systemic infections involving internal organs; or administration of antifungal therapy within 3 months prior to the study.

### 2.4 Antifungal susceptibility test

Antifungal susceptibility tests were performed to evaluate sensitivity to amphotericin B, itraconazole, fluconazole, posaconazole, voriconazole, anidulafungin, griseofulvin, and terbinafine (Sigma, St. Louis, MO, USA). According to the CLSI (Clinical and Laboratory Standards Institute) M27-E4 protocol (Wayne, 2017), yeast, yeast-like, and dimorphic fungi inocula were adjusted to a concentration of approximately 1–5×104 CFU/mL and introduced into prepared 96-wells. Minimum inhibitory concentrations (MIC) were recorded after 24 h of incubation at 35°C. According to CLSI M38-A3 protocol (Pa, 2017), dermatophyte fungi (including Trichophyton rubrum and Trichophyton tonsurans) were adjusted to a concentration of approximately 1–3×103 CFU/mL and non-dermatophyte fungi (including Fusarium solani, Fonsecaea pedrosoi, Microsphaeropsis arundini, and Paecilomyces lilacinus) were adjusted to a concentration of approximately 0.4–5×104 CFU/mL. The MICs of incubation were recorded at 35°C for 96 h for dermatophyte fungi an 46–50 h for F. solani, F. pedrosoi, M. arundini, P. lilacinus, and Sporothrix schenckii (Mahmoudi et al., 2016). The MIC of AmB was defined as the lowest concentration that resulted in complete growth inhibition. For other antifungal agents, the MIC was defined as the lowest concentration that achieved a 50% (for yeast, yeast-like, and dimorphic fungi inocula) or 80% (for dermatophyte fungi) decrease in growth compared with that in the drug-free control. To ensure optimal results, the susceptibility of each isolate was reassessed. Candida parapsilosis (ATCC 22019) and Trichophyton interdigitale (ATCC MYA 4439) were utilized as quality control strains for susceptibility testing. The MIC range and geometric mean (GM) for multiple strains were calculated.

### 2.5 Statistical analysis

Data were analyzed using the SPSS version 20.0 software (IBM Corp., Armonk, NY, USA). The medians and interquartile ranges were calculated using descriptive statistics.

## 3 Results

### 3.1 Clinical features of patients with primary subcutaneous mycoses

The clinical features and predisposing factors of patients with primary subcutaneous mycoses are summarized in **Table 1**. The male-to-female infection ratio was 1:0.83, suggesting a slightly higher prevalence in male patients. The median age of the patients was 60.6 (range, 14–82) years. The median duration of the primary subcutaneous mycoses infections was 5 months, with a range of 3–240 weeks between symptom onset and diagnosis. Trauma was the most common predisposing factor (n = 18, 54.5%), followed by tinea in other regions (n = 9, 27.3%). Other associated conditions include type 2 diabetes (n = 7, 21.2%), breast cancer (n = 2, 6.1%), systemic corticosteroid use (n = 1, 3%), and *CARD9* deficiency (n = 1, 3%). Topical corticosteroid treatment for primary disorders such as eczema (n = 4, 12.1%) was also a significant inducer of deep fungal infections. One case of primary subcutaneous mycoses was caused by exposure to the pesticide *Paecilomyces lilacinus*. Furthermore, five cases (15.2%) had no identifiable risk factors.

**Table 1:** Clinical features of patients with primary subcutaneous mycoses (n = 33).

Characteristics	n (%)
Sex	
Male	18 (54.5)
Female	15 (45.5)
Age (years)	
0–39	3 (9.1)
40–59	8 (24.2)
≥60	22 (66.7)
Median (range)	60.6 (14–82)
Duration, months	
≤3	9 (27.3)
>3, ≤6	9 (27.3)
>6	15 (45.5)
Median (range)	5 (0.75–240)
Predisposing factors	
Trauma	18 (54.5)
Previous tinea	9 (27.3)
Topical corticosteroids	4(12.1)
Pesticide spraying	1 (3.0)
Type 2 diabetes	7 (21.2)
Breast cancer	2 (6.1)
Systemic corticosteroids	1 (3.0)
*CARD9* deficiency	1 (3.0)
No associated factors	5 (15.2)
Initial location of skin lesion	
Arms	10 (30.3)
Hands	9 (27.3)
Legs	4 (12.1)
Feet	3 (9.1)
Face	5 (15.1)
Neck	1 (3.0)
Trunk	3 (9.1)
Scalp	1 (3.0)
Multiple sites	2 (6.1)
Type of lesion	
Nodules	17(51.5)
Papules	12 (36.4)
Plaques	20 (60.6)
Ulcers	9 (27.3)
No. of sites	
Single	21 (63.6)
Multiple	12 (36.4)

CARD9, Caspase recruitment domain family member 9.

**Figure 2:**
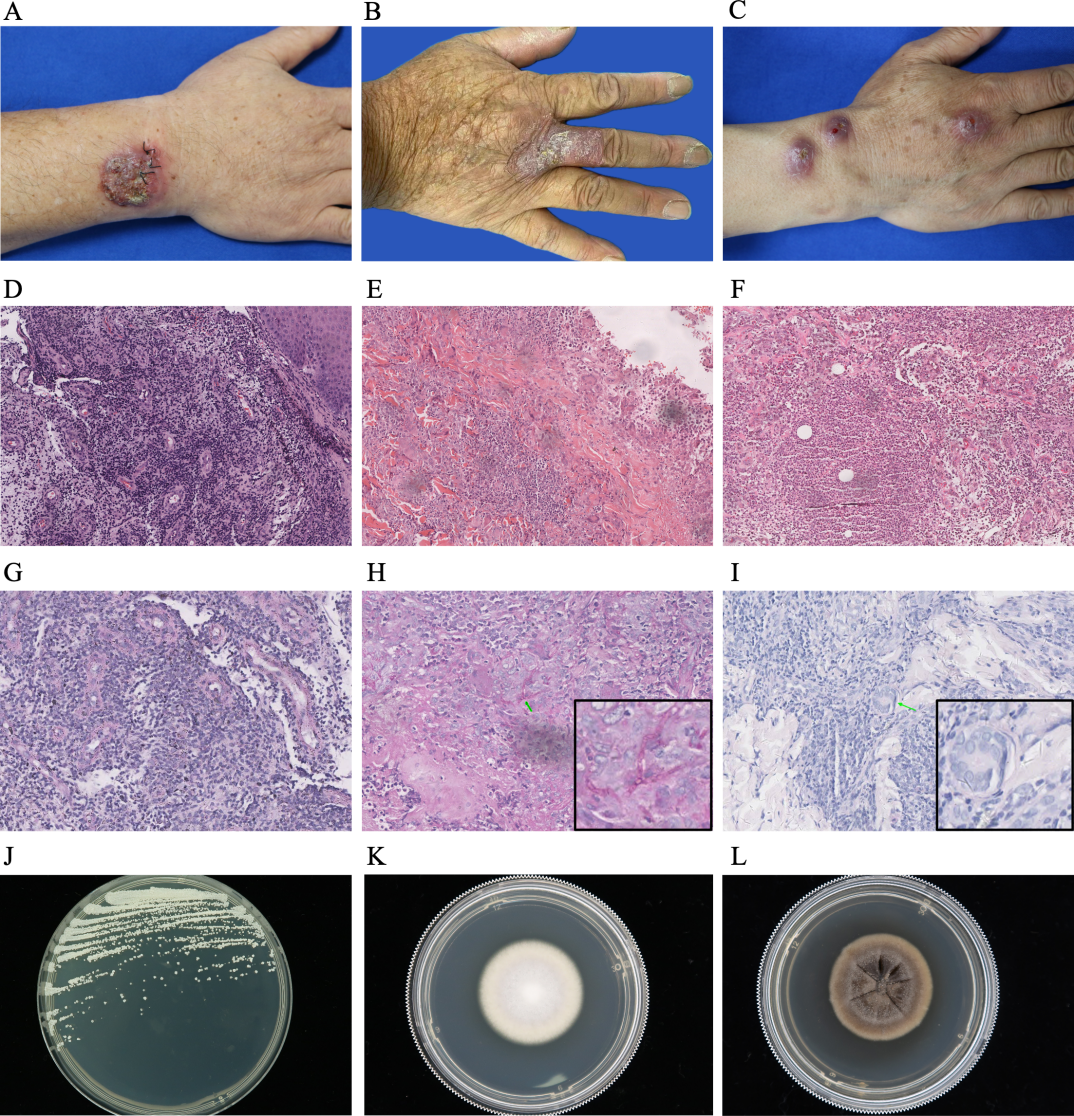
Diagnostic profile of clinical images, histopathological findings, PAS staining, and colony morphology of pathogenic fungi in patients with primary subcutaneous mycoses. **(A-C)** Skin lesions of patients with primary subcutaneous mycoses caused by *C. parapsilosis* **(A)**, *T. rubrum* **(B)**, and *S. schenckii* **(C)**. **(D–F)** Histopathological examination of patients with primary subcutaneous mycoses caused by *C. parapsilosis* **(D)**, *T. rubrum* **(E)**, and *S. schenckii* **(F)**, mainly manifested as infectious granulomas, reveal mild epidermal hyperplasia, histocytes, and multinucleated giant cells infiltrating the entire dermis, with a large number of visible infiltrating inflammatory cells (magnification: 100×). **(G–I)** PAS staining of samples from patients with primary subcutaneous mycoses caused by *C. parapsilosis* **(G)**, *T. rubrum* **(H)**, and *S. schenckii* **(J)** show negative results **(G)** or septate fungal hyphae **(H, I)** in the dermis (green arrow) (magnification: 200×). **(J–L)** Colony morphology of *C. parapsilosis* **(J)**, *T. rubrum* **(K)**, and *S. schenckii* **(L)**.

The arm (n = 10, 30.3%) was the most common site for skin lesions, followed by the hand (n = 9, 27.3%). These predilection sites were primary areas of vulnerability. The observed cutaneous manifestations included nodules (n = 17, 51.5%) (**Figure 2C**), papules (n = 12, 36.4%), plaques (n = 20, 60.6%) (**Figures 2A, B**), and ulcers (n = 9, 27.3%). Among the patients, 21 (63.6%) had a single lesion, whereas 12 (36.4%) had multiple lesions.

### 3.2 Diverse manifestations in patients with primary subcutaneous mycoses

Histopathological examination of the specimens, primarily presenting as infectious granulomas, revealed mild epidermal hyperplasia with infiltration of histocytes and multinucleated giant cells throughout the dermis. This was accompanied by extensive inflammatory cell infiltration (**Figures 2D–F**). PAS staining showed varying results, including negative findings (**Figure 2G**), the presence of septate fungal hyphae (**Figures 2H, I**), or dissociated or clustered conidia within the dermis. Ziehl–Neelsen staining was negative for all skin biopsy samples.

Bacteriological and mycological tests were performed on all suspected cases, and 33 patients were diagnosed with deep fungal infections. The identification of pathogens from the mycology culture of skin tissues revealed distinct colonial morphologies for various mycological species. Among these 33 isolates, 15 presented yeast-like colonies on SDA with different colors, including white (**Figure 2J**), yellowish white, cream, or dark black with an olive green feathery margin. Ten isolates formed mold colonies on SDA and displayed various colors at the center, such as white (**Figure 2K**), pink, whitish gray, taupe, and black. Eight isolates grew readily on PDA; the colonies were initially cream-colored with a yeast-like wrinkled surface, and they gradually developed brown or black pigmentation (**Figure 2L**).

### 3.3 Pathogens isolated from patients with primary subcutaneous mycoses

**Figure 3:**
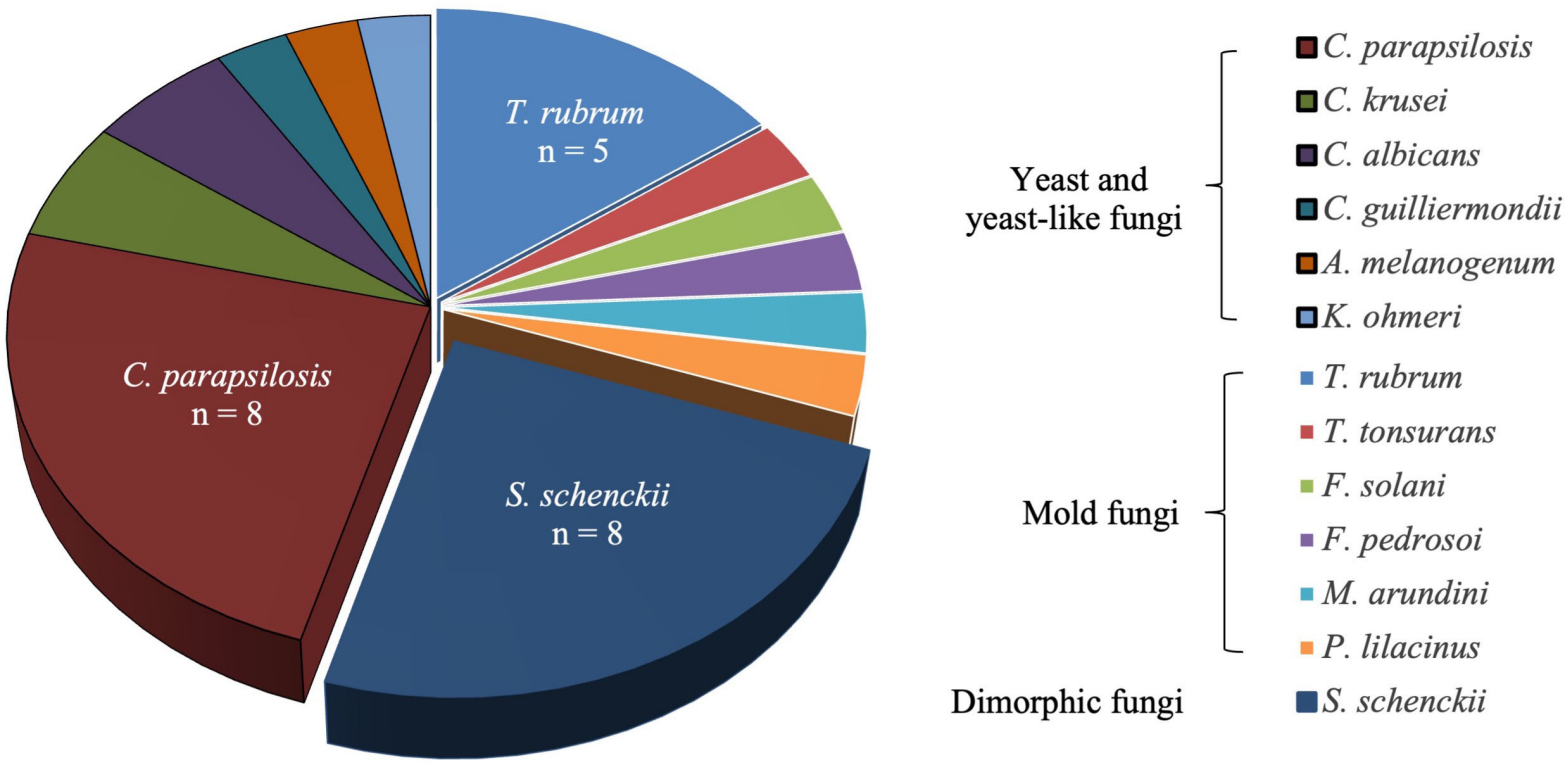
Species distribution of pathogens in patients with primary subcutaneous mycoses.

All isolated fungi were further confirmed through ITS region nucleotide sequencing. The predominate isolate was yeast and yeast-like fungi (n=15, 45.5%), and *C*. *parapsilosis* was the most common species (n = 8, 24.2%). Other yeast-like fungi included *C*. *krusei* (n = 2, 6.1%), *C*. *albicans* (n = 2, 6.1%), *C*. *guilliermondii* (n=1, 3.1%), *K*. *ohmeri* (n = 1, 3.1%), and *A*. *melanogenum* (n = 1, 3.1%). The second group consisted of mold fungi (n=10, 30.3%), and *T*. *rubrum* was the most prevalent species (n = 5, 15.2%), followed by *F*. *solani* (n = 1, 3.1%), *M*. *arudini* (n = 1, 3.1%), *T*. *tonsurans* (n = 1, 3.1%), *F*. *pedrosoi* (n = 1, 3.1%), and *P*. *lilacinus* (n = 1, 3.1%). The final group included dimorphic fungi and was represented solely by *S*. *schenckii* (n = 8, 24.2%) (**Figure 3**). Thus, a significant diversity of fungal species was responsible for primary cutaneous mycoses.

### 3.4 Antifungal medication sensitivity patterns of isolates from patients with primary subcutaneous mycoses

The ranges and geometric means of the MICs of eight antifungal agents for the pathogens in patients with primary deep cutaneous mycoses are summarized in **Table 2**. Most yeast and mold fungi were sensitive to azoles; however, some isolate strains exhibited resistance to fluconazole (MIC >8 µg/mL). *P*. *lilacinus* had a higher MIC value for itraconazole, suggesting potential resistance; however, it was represented by only one strain, and the rough data may not have been entirely precise. In contrast to the azole drugs, the MICs of amphotericin B and griseofulvin were higher for nearly all clinical isolates. Moreover, compared with the results of previous studies, our results showed that all clinical isolates exhibited relatively high MICs for terbinafine. Among the eight clinical isolates of *S*. *schenckii*, the MICs of antifungal agents were higher than those for the yeast and mold fungi. The novel antifungal drug, anidulafungin, had low MIC values for all clinical isolates, indicating high *in vitro* antifungal sensitivity. However, the breakpoint for *S*. *schenckii* and mold fungi has not yet been determined using the Standard Clinical and Laboratory Standards Institute M38 method.

**Table 2:** Minimum inhibitory concentrations (µg/mL) of eight drugs in antifungal susceptibility tests for pathogens of patients with primary subcutaneous mycoses.

Pathogens Antifungals	Fluconazole		Itraconazole		Posaconazole		Voriconazole		Terbinefin		Amphotericin B		Griseofulvin		Anidulafungin	
	Minimum inhibitory concentrations (ug/mL)															
	Range	GM	Range	GM	Range	GM	Range	GM	Range	GM	Range	GM	Range	GM	Range	GM
Yeast and yeast-like fungi																
*C. parapsilosis* (n = 8)	0.125–4	0.70	0.0313–0.25	0.136	0.0313–0.5	0.14	0.0313–0.0625	0.04	0.5–16	3.36	0.25–8	1.189	8–64	24.6	0.0078–2	0.16
*C. krusei* (n = 2)	8–16	11.31	0.25–0.5	0.35	0.125–0.5	0.25	0.063–0.25	0.125	2–8	4	0.25–1	0.5	32–64	45	0.063–0.125	0.08
*C. albicans* (n = 2)	1–2	1.4	0.5	0.5	0.125–0.25	0.17	0.0313–0.25	0.08	4–16	8	1–4	2	8–32	16	0.25–0.5	0.35
*C. guilliermondii* (n = 1)	2	–	0.25	–	0.25	–	0.031	–	2	–	0.25	–	32	–	1	–
*A. melanogenum* (n = 1)	8	–	0.25	–	0.5	–	0.031	–	1	–	8	–	16	–	0.125	–
*K. ohmeri* (n = 1)	2	–	0.25	–	0.125	–	0.063	–	2	–	0.25	–	64	–	0.031	–
Mold fungi																
*T. rubrum* (n = 5)	2-16	4.5	0.0313–0.25	0.07	0.0625–0.25	0.11	0.0313–0.125	0.05	1–8	2	8–16	9.1	0.125–2	0.37	0.0625–0.25	0.125
*T. tonsurans* (n = 1)	8	–	0.125	–	0.125	–	0.063	–	2	–	0.5	–	0.5	–	0.125	–
*F. solani* (n = 1)	8	–	2	–	1	–	0.5	–	4	–	16	–	32	–	4	–
*F. pedrosoi* (n = 1)	0.5	–	0.125	–	0.125	–	0.031	–	0.5	–	0.25	–	2	–	0.125	–
*M. arundini (n = 1)*	8	–	0.125	–	0.125	–	0.125	–	4	–	4	–	32	–	0.5	–
*P. lilacinus* (n = 1)	16	–	4	–	1	–	0.125	–	8	–	8	–	0.25	–	0.031	–
Dimorphic fungi																
*S. schenckii* (n = 8)	16–32	17.4	2–4	3.08	1–4	2	2–4	3.36	4–16	7.33	2–8	5.18	4-32	8	0.25–2	0.7

GM, geometric mean.

### 3.5 Therapeutic management and outcomes

Thirty-two patients received systemic antifungals for a median duration of 6 (range, 3–36) months, based on the results of drug sensitivity tests. The treatments included azoles (n = 32, 97.0%), primarily itraconazole (n = 31, 94.0%) and posaconazole (n=1, 3.0%), as well as allylamines (n = 5, 15.6%), specifically terbinafine (n = 5, 15.6%) (**Table 3**). Among these 32 patients, 6 were treated with a combination of itraconazole and terbinafine. Additionally, one patient with *CARD9* deficiency caused by *T*. *tonsurans* switched from voriconazole to posaconazole. One patient with a single forehead plaque caused by *K*. *ohmeri* was successfully treated using complete surgical resection.

**Table 3:** Treatment regimens and outcomes of patients with primary subcutaneous mycoses.

Variable	Number of patients
**Antifungal treatment**	32/33
Azoles	32/32
Itraconazole	31/32
Posaconazole	1/32
Allylamines	5/32
Terbinafine	5/32
Combination therapy*	6/32
Median duration (range), months	6 (3–36)
**Surgical treatment**	1/33
Outcome	
Complete response	30/33
Relapse	3/33

*Itraconazole combined with terbinafine.

The skin lesions subsided in all patients, resulting in clinical cure. Long-term follow-up is essential for patients with deep dermatophytosis. However, three patients experienced relapses within 2 years after clinical cure. The relapse was caused by *T*. *tonsurans* in the patient with *CARD9* deficiency, *F*. *pedrosoi* in a patient without any predisposing factors, and *P*. *lilacinus* in a patient after reexposure to pesticide spraying.

## 4 Discussion

Primary subcutaneous mycoses are a rare form of deep fungal infections that extend throughout the dermis and subcutis, affecting both immunocompromised and immunocompetent patients (Arenas et al., 2012; La Hoz and Baddley, 2012; Elgart, 2014; Carrasco-Zuber et al., 2016). The nonspecific clinical manifestations, lack of efficient diagnostic methods, and diversity of pathogenic fungi complicate the diagnosis of primary subcutaneous mycoses (Quindós et al., 2012; Gonzalez Santiago et al., 2014). Consequently, most reports in the literature have been in the form of individual cases or case series (Tessari et al., 2010; Kim et al., 2012, 2015; Tsai et al., 2017; Veríssimo et al., 2022; Hsu and Lee, 2023). In the present study, we examined 33 patients with primary subcutaneous mycoses caused by 13 opportunistic or non-opportunistic pathogens, contributing to the understanding of potential pathogenic fungi. Trauma and immune deficiency, including chemotherapeutic agents, malignancies, transplant anti-rejection drugs, AIDS, and *CARD9* deficiency, are the main triggers for the onset of subcutaneous mycoses (Tessari et al., 2010; Kershenovich et al., 2017; Bertin et al., 2024). Our study revealed that trauma was the most common predisposing factor, which was consistent with previous studies, followed by immune deficiency, which was substantially less common than previously reported (Kim et al., 2012, 2015; Tsai et al., 2017; Hsu and Lee, 2023). The reason for this discrepancy is that the eruptions in patients with primary subcutaneous mycoses were exclusively limited to the cutaneous and subcutaneous tissues, which are closely related to trauma. In contrast, patients with subcutaneous mycoses with systemic involvement, which is typically associated with immune deficiency, were excluded from this study.

The fungi responsible for primary subcutaneous mycoses are usually related to the geographical location, and the diversity of the fungal species involved is fairly limited. In our study, we analyzed 33 cases of primary subcutaneous mycoses caused by 13 different pathogens that were categorized as yeast and yeast-like fungi, mold fungi, and dimorphic fungi. First, six types of yeast and yeast-like fungi were identified, including *C*. *parapsilosis*, *C*. *krusei*, *C*. *albicans*, *C*. *guilliermondii*, *A*. *melanogenum*, and *K*. *ohmeri*. Among these, *C*. *parapsilosis* was the most common pathogen (8/15, 53.3%) in yeast and yeast-like infections. Over the past decade, infections caused by *C*. *parapsilosis* have risen dramatically (van Asbeck et al., 2009). This pathogen is widely found in nature and commonly exists as a human commensal, especially in the subungual space of human hands. Its pathogenicity is generally limited by an intact integument. The close association between *C*. *parapsilosis* infection and trauma is supported by its presence in approximately 80% of cases in our study. Second, the pathogenic mold fungi identified in this study were *T*. *rubrum*, *T*. *tonsurans*, *F*. *solani*, *F*. *pedrosoi*, *M*. *arudini*, and *P*. *lilacinus*, of which *T*. *rubrum* was the most prevalent (5/10, 50%). *T*. *rubrum* is a well-known pathogen associated with superficial infections, such as tinea pedis, onychomycosis, and tinea corporis. In immunosuppressed hosts, it can lead to subcutaneous dermatophytosis by penetrating the skin barrier (Kershenovich et al., 2017). Our findings indicate that the rate of immunosuppression among patients with mold fungi infections was higher than that among patients with yeast and yeast-like fungi (13% vs. 50%) and dimorphic fungi (12.5% vs. 50%) infections. Third, the only dimorphic fungi noted in this study was *S*. *schenckii.* Most patients with *S*. *schenckii* infections had a clear history of contact with environmental sources, such as flowers, wood splinters, soil, and vegetation (n = 5, 62.5%). This finding aligns with those of previous studies (Li et al., 2023) and reinforces the association between exposure to specific environmental factors and the risk of *S*. *schenckii* infections.

Primary subcutaneous mycoses are frequently underdiagnosed because of limited laboratory facilities and examinations. The traditional detection techniques mostly include histopathology, serology, and skin tissue culture. Histopathologic analyses are nonspecific and typically manifest as suppurative necrosis, granulomatous inflammation, and pseudoepitheliomatous hyperplasia. Thus, histopathologic diagnosis is generally combined with special staining techniques, including PAS and GMS staining, which help visualize the fungal morphology, such as septate hyphae, yeast, and pigments (Gonzalez Santiago et al., 2014; Kwizera et al., 2020). Although positive special staining and serological results can support the diagnosis of subcutaneous mycoses, they do not reliably identify the specific pathogenic fungal species. Accurate species-level identification of clinical fungal isolates is crucial for formulating effective antifungal treatment regimens. Presently, skin tissue culture is considered the only reliable method for detecting and identifying fungal species, along with assessing their sensitivity to drugs (Seas and Legua, 2022). However, culture methods are often time-consuming and have a lower positivity rate, resulting in delayed patient care and even misdiagnosis. Recently, several studies have reported that the use of various molecular diagnostic techniques, such as restriction fragment length polymorphism analysis, DNA or RNA sequencing, dot blot hybridization, and oligonucleotide arrays, have helped address the current limitations in the diagnosis of subcutaneous mycoses (Seas and Legua, 2022; Friedman and Schwartz, 2023; Simonsen et al., 2023). These techniques exhibit high sensitivity for detecting skin tissues with low levels of pathogenic fungi and can facilitate the rapid identification of fungal species and isolates. Despite these advantages, the clinical application of these diagnostic methods is limited due to their cost, potential for false positive results, lack of guidance for treatment, and the need for high-quality skin tissue samples. Therefore, combining multiple detection methods is crucial in the diagnosis of primary subcutaneous mycoses.

With high recurrence rates, resistance to therapy, and possible complications such as squamous cell carcinoma, primary subcutaneous mycoses present a major therapeutic challenge in clinical practice (Cassalia et al., 2023). *In vitro* drug sensitivity tests play a crucial role in successful treatment (LaRocco, 1991). In this study, 32 patients received systemic antifungal therapy based on the results of drug sensitivity tests. One patient with a single, well-defined, small cutaneous lesion underwent complete surgical excision and experienced no recurrence during the 2-year follow-up. The therapeutic approach to primary subcutaneous mycoses should include not only systemic antifungal drugs but also other treatments, such as surgical excision and photodynamic therapy (Seas and Legua, 2022). Additionally, we observed an interesting phenomenon in a patient with a *CARD9* mutation caused by *T*. *tonsurans* who showed no response to voriconazole but responded well to posaconazole, despite the strain being susceptible to both drugs according to the drug sensitivity test. This discrepancy may be attributed to voriconazole impairing the functionality of polymorphonuclear neutrophils (PMNs) in immune cells, whereas posaconazole appears to activate PMN responses and enhances the generation of reactive oxygen species, formation of neutrophil extracellular traps, and degranulation (Farowski et al., 2016; Ries et al., 2019). Therefore, when patients with subcutaneous mycoses show resistance to antifungal agents, the immunosuppressive effect of these drugs should be considered. Individual therapeutic regimens must be formulated for patients with primary subcutaneous mycoses based on the clinical manifestations, host immune status, species of pathogenic fungi, and properties of the antifungal agent.

Surveillance after clinical cure is often overlooked but is distinctly important in the management of primary subcutaneous mycoses. In the present study, all patients underwent long-term follow-up, and three patients experienced relapses. The first patient, who had a *CARD9* mutation and *T*. *tonsurans* infection, reported mental stress, insomnia, and irregular eating habits, leading to a recurrence of the disease 8 months after the initial lesions completely resolved. *CARD9* deficiency can cause an impaired immune response to fungal infections and result in refractory severe infection or resurgence (Hu et al., 2022). In this case, immunodeficiency likely contributed to the recurrence. The second patient, who had *F*. *pedrosoi* infection, developed the same skin lesions 2 years after the initial clinical cure without any obvious triggers. We speculated that *F*. *pedrosoi* and its byproducts, such as melanin, could inhibit the host immune response or form special structures such as muriform cells that resist antifungal agents or evade immune cell killing, allowing for long-term latency in the body. The third patient, who had a *P*. *lilacinus* infection, experienced disease recurrence after reexposure to an insecticide containing live *P*. *lilacinus*. This patient’s medical history indicated that each relapse of primary subcutaneous mycoses was associated with the pesticide. Thus, addressing unhealthy lifestyles, enhancing immune function, ensuring complete removal of pathogenic fungi, and avoiding reexposure to these fungi could effectively reduce the risk of relapse in primary subcutaneous mycoses.

The present study had some limitations. First, this was a retrospective review involving a small number of cases from a single institution. Large-scale studies, such as multicenter analyses, are required for a more comprehensive understanding of primary subcutaneous mycoses in Shanghai. Second, we may have underestimated the number of cases, given that we only included patients with positive skin tissue culture results, which may have yielded false negatives. Therefore, further research should incorporate multiple diagnostic approaches, including molecular techniques, to enhance the diagnosis of primary subcutaneous mycoses.

## 5 Conclusion

This study highlights the considerable diversity among fungal species implicated in primary subcutaneous mycoses and underscores the complexities involved in their accurate diagnosis and management. The challenges associated with identifying clinical information and achieving rapid diagnosis remain a major hurdle in clinical practice. Moreover, therapeutic schemes and surveillance after clinical cure are distinctly important in the management process for this condition. This study contributes valuable insights that may facilitate advancements in the understanding and management of primary subcutaneous mycoses, ultimately leading to better prognoses for affected patients.

## Data Availability

The names of the repository/repositories and accession number(s) can be found below: https://www.ncbi.nlm.nih.gov/, SUB15165377.

## References

[B1] Arenas R. Moreno-Coutiño G. Welsh O. (2012). Classification of subcutaneous and systemic mycoses. Clin. Dermatol. 30, 369–371. doi: 10.1016/j.clindermatol.2011.09.006 22682183

[B2] Barros M. B. de Almeida Paes R. Schubach A. O. (2011). *Sporothrix schenckii* and sporotrichosis. Clin. Microbiol. Rev. 24, 633–654. doi: 10.1128/CMR.00007-11 21976602 PMC3194828

[B3] Bertin C. Sitterlé E. Scemla A. Fraitag S. Delliere S. Guegan S.. (2024). Deep cutaneous mycoses in kidney transplant recipients: diagnostic and therapeutic challenges. Med. Mycol. 62, myae001. doi: 10.1093/mmy/myae001 38228404

[B4] Bonifaz A. Vazquez-Gonzalez D. Perusquia-Ortiz A. (2010). Subcutaneous mycoses: chromoblastomycosis, sporotrichosis and mycetoma. JDDG 8, 619–627. doi: 10.1111/j.1610-0387.2010.07453 20529168

[B5] Brodell R. T. (2021). JAAD game changers: diagnosis of deep cutaneous fungal infections: correlation between skin tissue culture and histopathology. J. Am. Acad. Dermatol. 85, 801. doi: 10.1016/j.jaad.2021.04.007 39549850

[B6] Brown G. D. Denning D. W. Gow N. A. R. Levitz S. M. Netea M. G. White T. C. (2012). 4:165rv13. Hidden killers: human fungal infections. Sci. Transl. Med. 4, 165rv13. doi: 10.1126/scitranslmed.3004404 23253612

[B7] Carrasco-Zuber J. E. Navarrete-Dechent C. Bonifaz A. Fich F. Vial-Letelier V. Berroeta-Mauriziano D. (2016). Cutaneous involvement in the deep mycoses: a literature review. Part I-Subcutaneous Mycoses. Actas Dermosifiliogr. 107, 806–815. doi: 10.1016/j.ad.2016.05.017 27374381

[B8] Cassalia F. Gratteri F. Azzi L. Tosi A. L. Giordani M. (2023). Deep mycosis mimicking cutaneous squamous cell carcinoma. Dermatol. Rep. 16, 9782. doi: 10.4081/dr.2023.9782 PMC11216138 38957634

[B9] Elgart G. W. (2014). Subcutaneous (deep) fungal infections. Semin. Cutan. Med. Surg. 33, 146–150. doi: 10.12788/j.sder.0112 25577856

[B10] Farowski F. Cornely O. A. Hartmann P. (2016). High intracellular concentrations of posaconazole do not impact on functional capacities of human polymorphonuclear neutrophils and monocyte-derived macrophages *in vitro*. Antimicrob. Agents Chemother. 60, 3533–3539. doi: 10.1128/AAC.02060-15 PMC4879367 27021317

[B11] Friedman D. Z. P. Schwartz I. S. (2023). Emerging diagnostics and therapeutics for invasive fungal infections. Infect. Dis. Clin. North Am. 37, 593–616. doi: 10.1016/j.idc.2023.05.001 37532392

[B12] Gonzalez Santiago T. M. Pritt B. Gibson L. E. Comfere N. I. (2014). Diagnosis of deep cutaneous fungal infections: correlation between skin tissue culture and histopathology. J. Am. Acad. Dermatol. 71, 293–301. doi: 10.1016/j.jaad.2014.03.042 24836547

[B13] Guevara A. Pereira Siqueira N. Ferreira Nery A. da Silva Cavalcante L. Hagen F. Hahn R. C. (2021). Chromoblastomycosis in Latin America and the Caribbean: Epidemiology over the past 50 years. Med. Mycol. 60, myab062. doi: 10.1093/mmy/myab062 34637525

[B14] Hsu T. J. Lee C. H. (2023). Implantation mycoses and invasive fungal infections with cutaneous involvement in tropical Taiwan: an 11-year retrospective study of a medical center. J. Fungi (Basel) 9, 322. doi: 10.3390/jof9030322 36983490 PMC10052647

[B15] Hu A. Hu Z. Zou H. Zhang J. Zhang D. Wang H.. (2022). CARD9 in host immunity to fungal, bacterial, viral, and parasitic infections: an update. Front. Microbiol. 13. doi: 10.3389/fmicb.2022.1021837 PMC9682022 36439825

[B16] Kershenovich R. Sherman S. Reiter O. Huss S. R. Didkovsky E. Mimouni D.. (2017). A unique clinicopathological manifestation of fungal infection: a case series of deep dermatophytosis in immunosuppressed patients. Am. J. Clin. Dermatol. 18, 697–704. doi: 10.1007/s40257-017-0276-y 28389891

[B17] Kim M. S. Kim J. K. Lee M. W. Moon K. C. Kim B. J. Son S. W.. (2015). Epidemiology of deep cutaneous fungal infections in Korea, (2006–2010). J. Dermatol. 42, 962–966. doi: 10.1111/1346-8138.12968 26105506

[B18] Kim M. S. Lee S. M. Sung H. S. Won C. H. Chang S. Lee M. W.. (2012). Clinical analysis of deep cutaneous mycoses: a 12-year experience at a single institution. Mycoses 55, 501–506. doi: 10.1111/j.1439-0507.2012.02191.x 22487296

[B19] Kwizera R. Bongomin F. Lukande R. (2020). Deep fungal infections diagnosed by histology in Uganda: a 70-year retrospective study. Med. Mycol. 58, 1044–1052. doi: 10.1093/mmy/myaa018 32242631 PMC7657094

[B20] La Hoz R. M. Baddley J. W. (2012). Subcutaneous fungal infections. Curr. Infect. Dis. Rep. 14, 530–539. doi: 10.1007/s11908-012-0275-3 22811027

[B21] LaRocco M. (1991). Recent developments in antifungal susceptibility testing. Clin. Microbiol. Newsl. 13, 81–85. doi: 10.1016/0196-4399(91)90038-W

[B22] Li J. Mou J. Wang Y. Zhang Y. Mou P. (2023). Difference analysis of cutaneous sporotrichosis between different regions in China: a secondary analysis based on published studies on sporotrichosis in China. Ann. Transl. Med. 11, 180. doi: 10.21037/atm-23-448 36923077 PMC10009553

[B23] Mahmoudi S. Zaini F. Kordbacheh P. Safara M Heidari M. (2016). Sporothrix schenckii complex in Iran: Molecular identification and antifungal susceptibility. Med. Mycol. 54 (6), 593–599. doi: 10.1093/mmy/myw006 26933207

[B24] Nishikawa T. (2015). Dermatomycoses and medically important fungi that are necessary subjects of study for dermatology specialists – a personal experience. Med. Mycol. J. 56, J15–J21. doi: 10.3314/mmj.56.J15 25855023

[B25] Pa W. (2017). Reference Method for Broth Dilution Antifungal Susceptibility Testing of Filamentous Fungi. 3rd ed. CLSI standard M38 (Clinical and Laboratory Standards Institute (CLSI). Available at: https://clsi.org/standards/products/microbiology/documents/m38/.

[B26] Pappas P. G. Alexander B. D. Andes D. R. Hadley S. Kauffman C. Freifeld A.. (2010). Invasive fungal infections among organ transplant recipients: results of the Transplant-Associated Infection Surveillance Network (TRANSNET). Clin. Infect. Dis. 50, 1101–1111. doi: 10.1086/651262 20218876

[B27] Queiroz-Telles F. Esterre P. Perez-Blanco M. Vitale R. G. Guedes Salgado C. Bonifaz A. (2009). Chromoblastomycosis: an overview of clinical manifestations, diagnosis and treatment. Med. Mycol. 47, 3–15. doi: 10.1080/13693780802538001 19085206

[B28] Queiroz-Telles F. McGinnis M. R. Salkin I. Graybill J. R. (2003). Subcutaneous mycoses. Infect. Dis. Clin. North Am. 17, 59–85. doi: 10.1016/S0891-5520(02)00066-1 12751261

[B29] Quindós G. Eraso E. López-Soria L. M. Ezpeleta G. (2012). Invasive fungal disease: conventional or molecular mycological diagnosis? Enferm. Infecc. Microbiol. Clin. 30, 560–571. doi: 10.1016/j.eimc.2011.10.018 22206948

[B30] Ries F. Alflen A. Aranda Lopez P. Beckert H. Theobald M. Schild H.. (2019). Antifungal drugs influence neutrophil effector functions. Antimicrob. Agents Chemother. 63, e02409–e02418. doi: 10.1128/AAC.02409-18 30910895 PMC6535511

[B31] Seas C. Legua P. (2022). Mycetoma, chromoblastomycosis and other deep fungal infections: diagnostic and treatment approach. Curr. Opin. Infect. Dis. 35, 379–383. doi: 10.1097/QCO.0000000000000870 35942857

[B32] Simonsen J. K. Mose K. F. Kristensen L. Herlin L. K. (2023). Deep dermatophytosis in an immunocompetent adult with no prior history of skin disease. Med. Mycol. Case Rep. 39, 31–33. doi: 10.1016/j.mmcr.2023.02.001 36819736 PMC9929626

[B33] Tessari G. Naldi L. Piaserico S. Boschiero L. Nacchia F. Forni A.. (2010). Incidence and clinical predictors of primary opportunistic deep cutaneous mycoses in solid organ transplant recipients: a multicenter cohort study. Clin. Transpl. 24, 328–333. doi: 10.1111/j.1399-0012.2009.01071.x 19712084

[B34] Tsai W. C. Lee C. H. Wu W. M. Lin S. H. Yang Y. C. Cheng Y. W.. (2017). Cutaneous manifestations of subcutaneous and systemic fungal infections in tropical regions: A retrospective study from a referral center in southern Taiwan. Int. J. Dermatol. 56, 623–629. doi: 10.1111/ijd.13497 28295235

[B35] van Asbeck E. C. V. Clemons K. V. Stevens D. A. (2009). Candida parapsilosis: a review of its epidemiology, pathogenesis, clinical aspects, typing and antimicrobial susceptibility. Crit. Rev. Microbiol. 35, 283–309. doi: 10.3109/10408410903213393 19821642

[B36] Veríssimo C. Toscano C. Ferreira T. Abreu G. Simões H. Diogo J.. (2022). Invasive and subcutaneous infections caused by filamentous fungi: report from a Portuguese multicentric surveillance program. Microorganisms 10, 1010. doi: 10.3390/microorganisms10051010 35630453 PMC9145964

[B37] Wayne P. (2017). Reference Method for Broth Dilution Antifungal Susceptibility Testing of Yeasts. 4th ed (Clinical and Laboratory Standards Institute). Document M27-E4.

